# De-escalation, adequacy of antibiotic therapy and culture positivity in septic patients: an observational study

**DOI:** 10.5935/0103-507X.20160044

**Published:** 2016

**Authors:** Rafael Barberena Moraes, Julián Alberto Viteri Guillén, William Javier Castillo Zabaleta, Flavia Kessler Borges

**Affiliations:** 1 Intensive Therapy Service, Hospital de Clínicas de Porto Alegre, Universidade Federal do Rio Grande do Sul - Porto Alegre (RS), Brazil.; 2 Internal Medicine Service, Hospital de Clínicas de Porto Alegre, Universidade Federal do Rio Grande do Sul - Porto Alegre (RS), Brazil.

**Keywords:** Anti-bacterial agents/administration & dosage, Shock, septic/drug therapy, Intensive care units

## Abstract

**Objective:**

To evaluate the prevalence of antibiotic de-escalation in patients diagnosed with severe sepsis or septic shock at a public academic tertiary hospital and to evaluate antibiotic adequacy and culture positivity.

**Methods:**

The prevalence of antibiotic de-escalation, the adequacy of antibiotic treatment and the rates of culture positivity were analyzed in patients with severe sepsis and septic shock between April and December 2013 at an intensive care unit in a tertiary university hospital.

**Results:**

Among the 224 patients included in the study, de-escalation was appropriate in 66 patients (29.4%) but was implemented in 44 patients (19.6%). Among the patients who underwent de-escalation, half experienced narrowing of the antimicrobial spectrum. The mortality rate was 56.3%, with no differences between the patients with or without de-escalation (56.8% versus 56.1%; p = 0.999) nor in the length of hospital stay. Empirical antibiotic therapy was appropriate in 89% of cases. Microorganisms were isolated from total cultures in 30% of cases and from blood cultures in 26.3% of cases.

**Conclusion:**

The adequacy rate of empirical antibiotic therapy was high, reflecting an active institutional policy of monitoring epidemiological profiles and institutional protocols on antimicrobial use. However, antibiotic de-escalation could have been implemented in a greater number of patients. De-escalation did not affect mortality rates.

## Abstract (pt)

**Objetivo:**

Avaliar a prevalência de descalonamento antibiótico em pacientes com diagnóstico de sepse grave ou choque séptico em hospital acadêmico, público e terciário, além da adequação antibiótica e da positividade de culturas.

**Métodos:**

Foram analisadas prevalência de descalonamento, adequação antibiótica e positividade de culturas entre portadores de sepse grave e choque séptico, entre abril e dezembro de 2013, em uma unidade de terapia intensiva de um hospital universitário terciário.

**Resultados:**

Entre os 224 pacientes incluídos, o descalonamento era possível em 29,4% dos casos (66 pacientes), mas foi implementado em 19,6% deles (44 pacientes). Entre os pacientes que receberam descalonamento, metade foi por estreitamento de espectro antimicrobiano. A mortalidade foi de 56,3%, não havendo diferença entre pacientes com ou sem descalonamento (56,8% *versus* 56,1%; p = 0,999), assim como no tempo de internação. Terapia antimicrobiana empírica foi adequada em 89% dos casos. Houve isolamento de germe em 30% de todas as culturas e em 26,3% das hemoculturas.

**Conclusão:**

A taxa de adequação antibiótica empírica foi alta, refletindo ativa política institucional de monitorização do perfil epidemiológico e protocolos institucionais de uso de antimicrobianos. No entanto, o descalonamento antimicrobiano poderia ter sido maior do que o registrado. O descalonamento não impactou mortalidade.

## INTRODUCTION

Infectious diseases are responsible for more than a third of hospital admissions and are one of the most prevalent causes of admission to intensive care units (ICU) worldwide.^(1)^ Sepsis has a high global mortality rate.^(2)^ For patients who have been diagnosed with sepsis and admitted to the ICU, mortality rates can reach up to 60% in low-income countries.^(3)^ Multi-drug-resistant organisms have become more frequent while the development of new antimicrobial agents has slowed.^(4)^ Therefore, optimizing the use of antibiotics is key to stemming the increasing rates of multi-drug-resistant infections. The selection of adequate antibiotics at the time of a sepsis diagnosis must be based on the microbiological profile and local antimicrobial susceptibility. In this context, the early use of broad-spectrum antibiotics, optimization of the dose, route, and duration of antibiotic administration, and adjustment or discontinuation of antibiotic therapy as soon as possible based on microorganism susceptibility are essential.^(5)^

Two important approaches for optimizing the treatment of septic patients and decreasing costs are de-escalation and monitoring the adequacy of antibiotic therapy. Antibiotic de-escalation is defined as narrowing of the antimicrobial spectrum based on the susceptibility of the pathogen, thereby decreasing the possibility of bacterial resistance. The antibiotic spectrum must be narrowed as soon as possible, considering the clinical condition of the patient, the pathogens identified in cultures and the sensitivity profile obtained from the antibiogram. When no evidence of bacterial infection is present, antibiotic therapy must be suspended.^(6)^

The literature recommends antibiotic de-escalation as an appropriate practice, which leads to cost reductions and decreased bacterial resistance. However, Silva et al. conducted a meta-analysis and found no direct evidence that antibiotic de-escalation is safe and effective in adults with severe sepsis and septic shock based on observational studies.^(7)^

Two more recent studies suggest that antibiotic de-escalation is efficient and safe. Garnacho-Montero et al. found reduced in-hospital and 90-day mortality rates in an observational study of patients with severe sepsis and septic shock.^(8)^ Leone et al. conducted a multicenter non-inferiority clinical trial and found similar mortality rates in patients undergoing antibiotic de-escalation and in patients for whom the initial treatment was maintained.^(9)^ Despite these divergent results, several authors support the use of de-escalation as an important strategy against the development of antimicrobial resistance.^(10)^ Similarly, the adequacy of antibiotic therapy is likely related to decreased mortality from sepsis.^(11,12)^ To achieve high rates of antibiotic adequacy, knowledge of the local antimicrobial resistance profile is required.

These data are widely described in studies from the United States, Europe and Australia;^(5,6,8,9)^ however, similar results have not been found in developing countries where mortality rates for sepsis are much higher. Therefore, the main objective of this study was to evaluate the prevalence of antibiotic de-escalation in patients with severe sepsis and septic shock who were admitted to a tertiary hospital in southern Brazil. The secondary outcomes were the adequacy of empirical antibiotic therapy and culture positivity rates in patients who were diagnosed with sepsis or septic shock and admitted to a Brazilian public hospital.

## METHODS

Since 2013, the Hospital de Clínicas de Porto Alegre (HCPA) monitors the care of patients with severe sepsis and septic shock by prospectively collecting care data using a questionnaire standardized by the Latin American Sepsis Institute (Instituto Latino Americano de Sepse - ILAS).^(13)^ This observational cohort study included all patients with severe sepsis and septic shock who received care at the HCPA and who were admitted to the ICU or the vascular emergency unit between April and December 2013 according to the ILAS database. All patients under the age of 18 and patients who had decided to withhold life support were excluded.

Severe sepsis was defined as sepsis and hypotension (systolic blood pressure < 90mmHg or mean blood pressure < 60mmHg) and evidence of at least one of the following organic dysfunctions: an altered level of consciousness, a lactate level > 2mmol/L, diuresis < 0.5mL/kg in 6 hours, a partial pressure oxygen and fraction of inspired oxygen (PaO_2_/FiO_2_) ratio < 300 or thrombocytopenia < 100,000∕µL. Patients were considered to have septic shock if they required vasopressors despite adequate fluid resuscitation, i.e., at least 20mL crystalloid solution per kg of weight.^(14)^

The HCPA is a public tertiary university hospital. Approximately 95% of patients are seen through the Unified Health System (Sistema Único de Saude - SUS), and this hospital is the regional reference center for high-complexity care. The ICU is composed of 33 beds and has 7 nurses and 1 nursing technician for every 2 patients. The vascular emergency unit had 9 beds, 1 nurse and 3 nursing technicians. The hospital has approximately 600 inpatient beds for adults. The HCPA has a Hospital Infection Control Commission (Comissão de Controle de Infecção Hospitalar - CCIH), which defines local policies for antimicrobial use. The CCIH monitors the local profile of antimicrobial resistance, periodically updating local protocols for the empirical use of antimicrobials. All patients included in this study received empirical antibiotic therapy following the institutional protocols developed by CCIH.^(15)^ Antibiotic therapy was prescribed by the assistant physician, who discussed the therapeutic regimen with CCIH consultants.

All collected cultures and prescribed antibiotic treatments were searched in the electronic system (AGHWEB-HCPA), and microbiological tests and antibiotic drugs were recorded for each patient. The information included the number of requested cultures, the number of positive cultures, microorganisms, infectious foci and the type of antibiotics used. Most cultures were obtained through conventional culturing and qualitative methods (urine culture, abdominal and pleural effusions, spinal fluid, sputum and tracheal aspirate); sputum cultures cannot be quantified, whereas tracheal aspirate analysis is quantitative in nature. Blood cultures and ascitic fluid analysis were performed using an automated method (BACTALERT), generating qualitative results.

The medical records of the patients were reviewed by two independent evaluators (third-year residents of the Internal Medicine Service) and, subsequently, by two senior evaluators (physicians with a postgraduate degree, namely a PhD in medicine, and professors of the Intensive Therapy Service and Internal Medicine). The senior evaluators assessed the prevalence of de-escalation and the adequacy of treatment. Disagreements were resolved by a consensus between the four evaluators.

Antibiotic de-escalation was defined as narrowing of the antimicrobial spectrum (antibiotics active against a smaller number of bacterial species) based on the susceptibility of the pathogen.^(3)^ Therefore, we defined de-escalation as follows: (1) narrowing of the antibiotic spectrum based on the antibiogram, (2) reduction in the number of antimicrobial agents, and (3) discontinuation of the antibiotic (3^rd^ to 5^th^ day) or initial discontinuation of treatment (before the third day) even with negative cultures.^(16)^ The adequacy of the initial antibiotic therapy was based on the sensitivity profile of the isolated microorganisms according to the infectious focus. In cases of polymicrobial biota, the absence of microorganisms or the isolated growth of microorganisms with no relation to the infectious focus, only the infectious focus was used as adequacy criteria. In this case, prescription of an antimicrobial regimen to cover the infectious focus was considered adequate according to institutional CCIH standards.^(15)^ Treatment maintenance was defined as the maintenance of the initial treatment over the first 7 days of sepsis or septic shock. Broadening of the antimicrobial spectrum or escalation was observed in cases that required antibiotics active against a larger group of bacterial species when compared with the initial regimen despite the presence of positive cultures.

The patients were evaluated for the use of mechanical ventilation in the first 24 hours after the diagnosis of sepsis, the length of hospital stay and the in-hospital mortality rate.

The evaluators identified all patients for whom the antimicrobial regimen could have been de-escalated according to the microbiological tests, i.e., the antibiogram showed antibiotics with a narrower spectrum in culture that were compatible with the infectious focus.

The data were analyzed using the Statistical Package for the Social Sciences (SPSS) (IBM) version 18.0. A descriptive analysis was performed, and the data are expressed as means and standard deviations or medians and interquartile ranges of the continuous variables and the absolute and relative frequencies of the categorical variables. To identify the differences between the variables in the comparison between antibiotic de-escalation and escalation maintenance, the chi-squared and Fisher's exact tests were used for the categorical variables, and the Mann-Whitney test was used for the continuous variables. A p value of 0.05 indicated statistical significance.

Considering a de-escalation prevalence of 23%,^(5)^ a confidence interval (CI) of 95% and an acceptable difference of 5% (18% - 28%), we calculated a sample size of 225 patients who were prescribed antibiotic therapy to complete the study.

The project was approved by the Research Ethics Committee of the institution under number 140300. An informed consent form was waived by the committee because this study was observational and used data from medical records only.

## RESULTS

A total of 224 patients were included in this study. The clinical and epidemiological characteristics of the cohort are shown in table 1. The data show a high prevalence of mechanical ventilation and a high mortality rate similar to that observed in public hospitals in Brazil.^(17)^ The mortality rate of the cohort was 56.3%, with no differences in the mortality rates between the patients who underwent antibiotic de-escalation and the patients who did not (56.8% versus 56.1%; p = 0.999).

**Table 1:** Clinical and epidemiological characteristics of the study cohort

Variable	Without de-escalation (N = 180)	With de-escalation (N = 44)	p value
Age (years)	59 ± 16	62 ± 16	> 0.05
Gender			< 0.05
Male	97 (54)	30 (68)	
Focus of sepsis			< 0.05
Pneumonia-empyema	104 (58)	22 (50)	
Urinary tract infection	11 (6.1)	3 (6.8)	
Acute abdominal infection	35 (19)	5 (11.4)	
Meningitis	2 (1.1)	1 (2.3)	
Skin - soft parts	8 (4.4)	3 (6.8)	
Surgical wound infection	-	2 (4.5)	
Catheter-related bloodstream infection	3 (1.7)	2 (4.5)	
Endocarditis	1 (0.6)	3 (6.8)	
Prosthesis-related infection	2 (1.1)	-	
Multiple foci	1 (0.6)	-	
Other infections	13 (7.2)	3 (6.8)	
APACHE II	25 ± 7.6	26 ± 8.5	> 0.05
SOFA	7.3 ± 3.8	7.9 ± 3.6	> 0.05
Positive cultures			< 0.05
Blood cultures	35 (19)	16 (42)	
Sputum	20 (12)	15 (34)	
Urine culture	19 (10.6)	10 (25)	
Abdominal effusions	13 (7.8)	4 (9.1)	
Other material	11 (6.1)	6 (16)	
Microorganism identified in the culture			< 0.05
*Staphylococcus aureus*	12 (7)	7 (4.2)	
Coagulase-negative *staphylococci*	8 (4.8)	5 (3)	
*Streptococcus pneumoniae*	8 (4.8)	4 (2.4)	
*Escherichia coli*	18 (10.8)	6 (3.6)	
*Klebsiella pneumoniae*	10 (6)	5 (3)	
*Enterococcus sp*	9 (5.4)	4 (2.4)	
*Acinetobacter sp*	1 (0.6)	-	
*Candida sp*	12 (7)	7 (4.2)	
*Pseudomonas Aeruginosa*	4 (2.4)	2 (1.2)	
*Proteus*	3 (1.8)	1 (0.6)	
Virus	4 (2.4)	1 (0.6)	
Mycobacteria	4 (2.4)	-	
Other	20 (12)	11 (6.6)	
Mechanical ventilation in the first 24 hours of sepsis	141 (78)	33 (75)	> 0.05
Adequate initial antibiotic treatment	158 (88)	42 (95)	> 0.05
Time until sepsis diagnosis since hospital admission (days)	6 ± 15.3	7.7 ± 8.7	> 0.05
Length of hospital stay (days)	19.5 [10 - 40]	21 [10 - 37]	> 0.05
Deaths	101 (56.1)	25 (56.8)	> 0.05

APACHE - Acute Physiology and Chronic Health Evaluation; SOFA - Sequential Organ Dysfunction Score. The results are expressed as the mean ± standard deviation, number (%) and median [25% - 75%].

**Figure 1:**
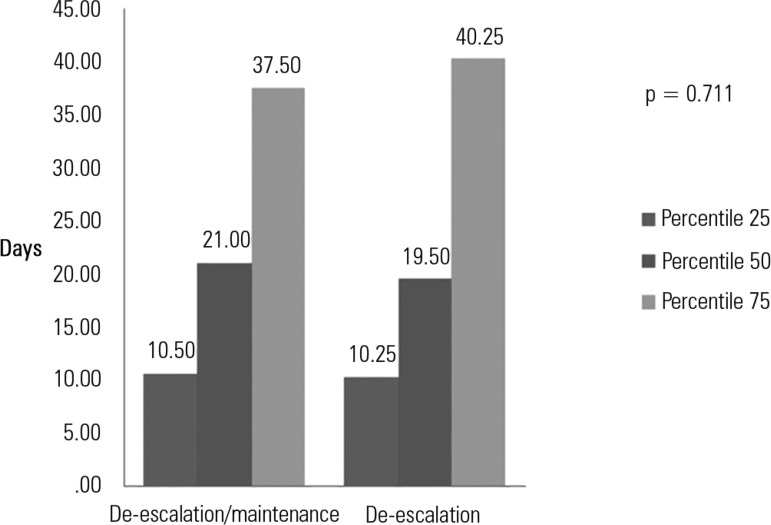
Length of hospital stay (days; 25^th^, 50^th^ and 75^th^ percentiles) for the patients who underwent escalation or maintenance of antibiotic treatment versus the patients who underwent de-escalation.

No differences were observed in the length of hospital stay between the patients who underwent antibiotic de-escalation and the patients who did not. Figure 1 shows similar results between the two groups (p = 0.711).

We identified 66 patients who could have undergone antibiotic de-escalation (29.4%); however, of the 224 patients included in the study, antibiotic treatment was maintained in 103 (46%) patients (95%CI 36.5 - 52.5), escalated in 77 (34.4%) patients (95%CI 28.4 - 40.8) and de-escalated in 44 (19.6%) patients (95%CI 14.8 - 25.2). Antibiotic de-escalation was implemented by narrowing the antimicrobial spectrum in 24 (54.5%) patients, reducing the number of antibiotics in 17 (38.5%) patients and discontinuing the antibiotic early in only 3 (7%) patients. In 200 (89.3%) patients, the initial empirical antibiotic treatment was adequate according to the CCIH guidelines of our institution (Table 2). A pulmonary focus of sepsis was predominant (126 patients, 56.3%), followed by acute abdominal infection (40 patients, 12.9%).

**Table 2:** De-escalation, adequacy of antibiotic treatment and culture positivity

Variable	N (%)	95%CI
Antibiotic treatment		
De-escalation	44 (19.6)	[14.8 - 25.2]
Narrowing of the antibiotic spectrum	24 (54.5)	
Reduction in the number of antibiotics	17 (38.6)	
Early discontinuation of antibiotic(s)	3 (6.8)	
Maintenance	103 (46)	[39.5 - 52.5]
Escalation	77 (34.4)	[28.4 - 40.8]
Possibility of de-escalation		
Yes	66 (29.4)	
No	158 (70.5)	

95%CI - 95% confidence interval.

A total of 506 cultures were collected from 224 patients, of which 151 were positive (30%). Those cultures included 201 blood cultures, 120 respiratory secretions, 104 urine cultures and 34 abdominal effusions with bacterial growth rates of 26.3% (51 samples), 29% (35 samples), 27.8% (29 samples) and 50% (17 samples), respectively. The prevalence of the isolated microorganisms is shown in table 1.

## DISCUSSION

This study found suboptimal de-escalation rates, high rates of empirical antimicrobial adequacy and low rates of positive blood cultures.

The prevalence of de-escalation varies widely in the literature, ranging from 10% to 70%.^(7-9,16,18)^ The prevalence in this study (19.6%), which was performed in a developing country at a center with a low rate of adherence to sepsis care bundles and a high mortality rate, is within the values reported in the literature.^(17)^ However, this rate could be higher, as described in previous studies that demonstrated rates up to 30%. Both the prevalence of de-escalation and the rate of antibiotic adequacy in this study are similar to those in a prospective observational study conducted in 24 ICUs in Spain, which found an adequacy rate of 91% and a de-escalation rate of 23%; however, de-escalation could have been performed in 39% of the patients.^(18)^

The reason for the lower prevalence of de-escalation in our sample was not analyzed in this study; however, the potential causes of this low prevalence are similar to those described in previous studies, including the fear of implementing de-escalation in patients with severe sepsis or septic shock given the severity of the cases. Another potential cause is the habit of not changing the treatment of patients whose condition is improving.^(5,8,10,16,18)^ In our cohort, de-escalation occurred through early discontinuation of the antibiotic in only 7% of cases; this type of de-escalation may be used to increase de-escalation rates and warrants further study. Several studies have demonstrated that antibiotic de-escalation is a safe and beneficial strategy.^(19-21)^ To optimize de-escalation rates, we should work to disseminate results from observational studies and expert opinions that suggest de-escalation is a safe and economical strategy, which can benefit both patients and care institutions. These benefits include less pressure on microbial selection, a reduced length of hospital stay and reduced hospital costs. The reduction in hospital costs is particularly relevant, especially in low-income countries, because de-escalation does not compromise treatment efficiency, thus improving the quality of care.^(16)^ Among the different strategies to increase de-escalation rates, we suggest a permanent dialogue through rounds or consults between physicians who prescribe antibiotics and the infection control committee members. Because this study was conducted at a teaching hospital, the de-escalation rates may be lower at other hospitals, particularly in developing countries not affiliated with academic centers or without proper structuring of the hospital infection commissions.

The adequacy of antibiotic therapy in this study was particularly high (89%). We believe that this is due largely to the intense interface between the prescribing physicians and the infection control commission. The CCIH at our hospital monitors the institutional microbiological profile and collaborates with other services to develop and update local protocols. Despite the high rates of antibiotic adequacy, mortality from septic shock at our institution remains high. This finding demonstrates that the adequacy of antibiotic therapy is part of a chain of events in the treatment of septic patients. To achieve mortality rates close to those found in developed countries, the adequacy of antibiotic therapy must include one of the following measures: antibiotic administration in the first hour of a diagnosis of severe sepsis, early recognition of these patients, use of adequate doses of antibiotics while remaining aware of the pharmacokinetic and pharmacodynamic changes in critical patients and adequate hemodynamic management.

Institutions with high rates of adherence to sepsis care bundles can reduce mortality from sepsis. A study of 2,120 patients at 10 private institutions in Brazil showed that multi-faceted interventions in severe sepsis and septic shock can be cost effective and reduce mortality rates even in developing countries.^(22)^

Therefore, institutions should monitor the quality of care provided to patients with severe sepsis and septic shock and relevant outcomes, such as mortality rates and length of hospital stay.

The rates of positive cultures were comparable to those in previous case series but lower than those in clinical trials.^(23)^ Optimization of culture collection procedures can benefit institutions and patients. To increase positivity rates, it is important to adopt a series of measures, including obtaining cultures, especially blood cultures, before the start of antibiotic treatment and using the correct collection technique, such as collecting the right volume and ensuring that individuals collecting the cultures wash their hands properly. Cultures with false positives arising from incorrect culture collection procedures lead to increased hospital costs. The use of specialized teams to collect these cultures and the continued training of those collecting cultures are potentially cost-effective alternatives.^(23,24)^ The adequate collection of cultures enables increased de-escalation rates and subsequent benefits.

This study has several limitations. The evaluation of antibiotic de-escalation was performed retrospectively by reviewing the medical records of patients included in the ILAS database. To decrease the possibility of data misinterpretation, experienced reviewers analyzed the medical records independently, and disagreements were resolved by consensus. In addition, using the discontinuation of treatment before the third day as a de-escalation indicator may have overestimated the results because the suspension of antibiotic treatment may have been based on no evidence of infection rather than de-escalation. However, this situation was observed in only 3 patients. Because this study is retrospective, despite access to the patient records, prospective discussions with the prescribing physicians were not possible, which may limit the analysis of cases wherein the clinical picture suggested maintenance of the antimicrobial regimen even though the microbiological results suggested de-escalation. Moreover, external validity is another limitation because this study was single-center and performed in a public tertiary teaching hospital in southern Brazil.

## CONCLUSION

Even academic centers with high rates of empirical antibiotic adequacy can optimize the treatment of septic patients and reduce costs by increasing the prevalence of antibiotic de-escalation and culture positivity. These measures must be associated with other processes recommended for the treatment of patients with septic shock to ensure that developing countries attain mortality rates similar to those reported in developed countries.
